# Supplementary figures and images for: Diversity, recombination and misclassification in the family Geminiviridae: Insight from bioinformatics analysis
Source: PLoS One. 2026 Jan 2;21(1):e0338481. doi: 10.1371/journal.pone.0338481 (PMC12758780; doi:10.1371/journal.pone.0338481)

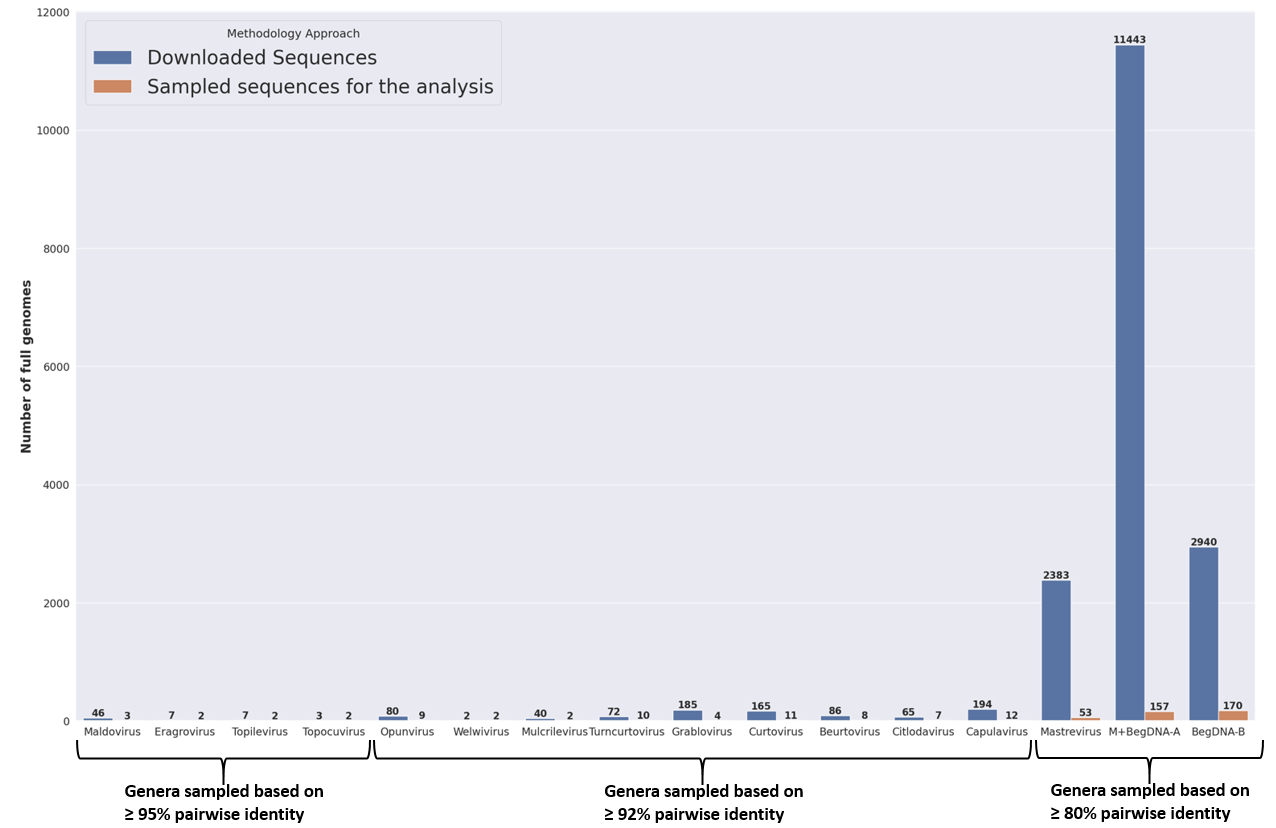

Supplement: S1 Fig — The bar chart depicted the retrieved and sampled nucleotide sequences across each genus. (TIF) [file pone.0338481.s001.tif]

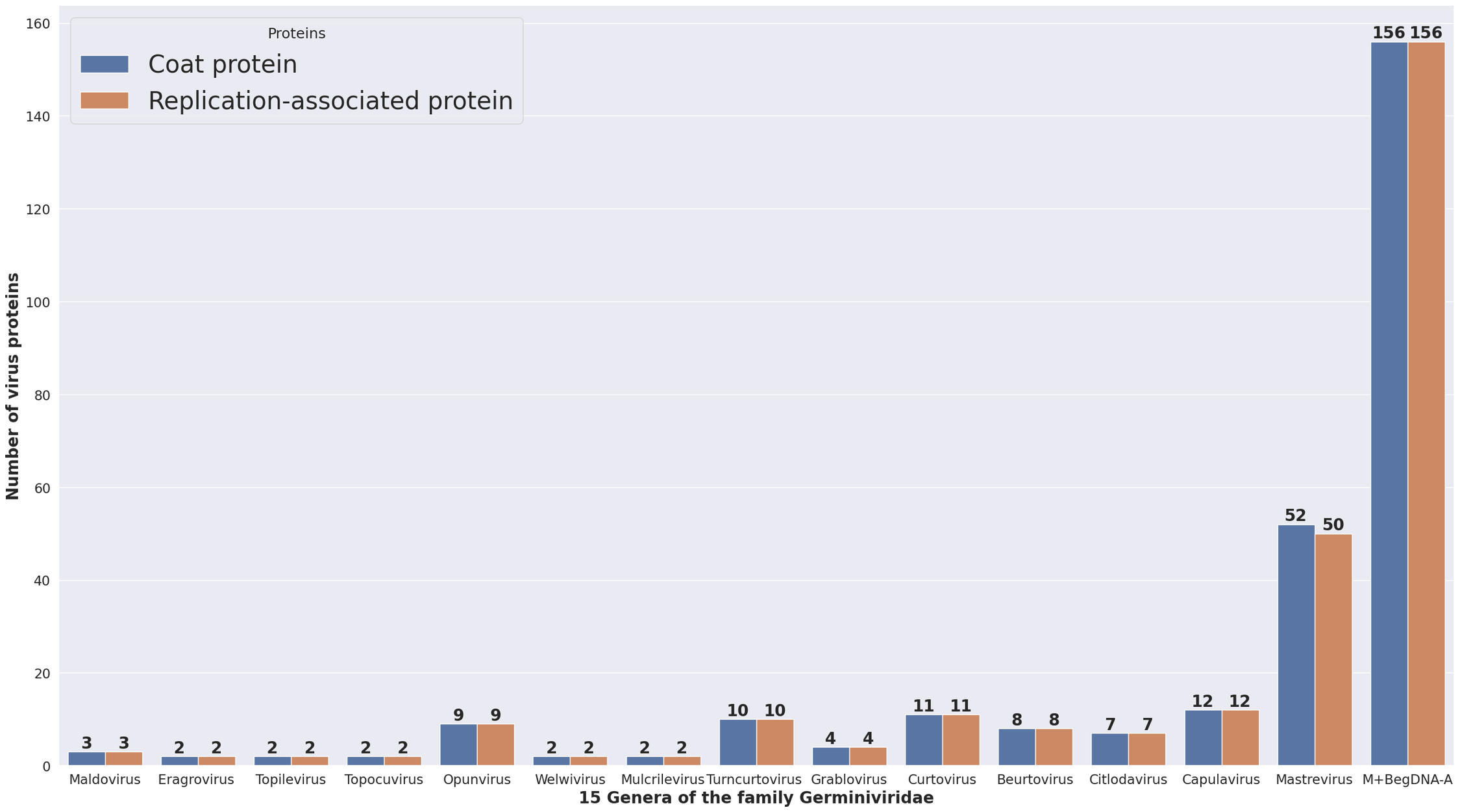

Supplement: S2 Fig — (TIF) [file pone.0338481.s002.tif]
