# Supplementary material for: Diversity, recombination and misclassification in the family Geminiviridae: Insight from bioinformatics analysis
Source: PLoS One. 2026 Jan 2;21(1):e0338481. doi: 10.1371/journal.pone.0338481 (PMC12758780; doi:10.1371/journal.pone.0338481)
Supplement: Attachment — Submitted filename: Response letter.pdf [file pone.0338481.s008.pdf]

September 28, 2025

Dr. Islam Hamim,

Academic Editor

PLOS ONE

Response to the reviewer comments for manuscript ID: PONE-D-25-15367R1

Dear Dr. Hamim,

We sincerely thank you and the esteemed reviewers for the constructive and insightful comments on our manuscript. We have carefully considered each suggestion and implemented the necessary revisions to improve the clarity, quality, and overall rigor of the work.

Below, we provide a point-by-point response to each comment. We hope this revised manuscript satisfactorily addresses all concerns and meets the standards of the journal.

Dr. Neil Arvin Bretana

Reviewer 1

Comment 1: L.52-56: Please cite the viral species responsible.

Response 1: We appreciate this comment, and the responsible species have been cited accordingly.

Change Made: Described in lines 52-60

Comment 2: L.107: The link https://doi.org/10.17504/protocols.io.j8nlkybjdg5r/v1 does not work.

Response 2: This observation is well appreciated. Truly, this is a reserve DOI because the protocol has not been published. The protocol publication is delayed to allow both editor and reviewers to review it, since the protocol is an integral part of this manuscript. As stated in the journal guideline, this reserve DOI will be automatically made public once the manuscript is published. As earlier included in the previous revision, this is the private link to access the protocol: https://www.protocols.io/private/9889AFB557F711F0B1B50A58A9FEAC02

Comment 3: L.169-170: I suggest changing the sentence to: "Additionally, the African continent had the highest number of countries where geminivirus infections are reported." Instead of: "Additionally, the African continent had the highest number of countries affected with geminivirus infections."

Response 3: This observation of avoiding potential bias is well appreciated and implemented accordingly.

Change Made: Described in lines 169-170

Reviewer 2

Comment 1: The authors may propose names for new virus isolates, but it is the ICTV's role to determine whether a new species should be formally recognized. Begomovirus spathoglottis 1 and 2 are introduced only in the Abstract and Results, with no prior mention in the Introduction and no references supporting their recognition as distinct species. The manuscript should explicitly

clarify that if the species status of Begomovirus spathoglottis 1 and 2 is not determined, these viruses do not represent officially recognized species. The proposed names "Maldovirus spathoglottis 1 and 2" do not constitute a formal species description; accordingly, they should not be italicized and should be written in lowercase, as italics are reserved for ICTV-recognized binomial species names. The heading "“Proposed new species within the genus Maldovirus" could be misleading. It would be clearer to indicate that these are proposed virus names rather than formal species. If biological evidence (e.g., fulfillment of Koch’s postulates) has been provided to support the designation of a new species, this should be clearly stated. Otherwise, the authors may consider either providing stronger evidence to justify species designation or clarifying that these are provisional virus names.

Response 1: Thank you for this thoughtful comment regarding the potentially misleading subtitle related to the proposed virus names for unclassified viruses. We have clarified that these proposed names have not yet been formally classified into species and that the formal recognition of new species is the responsibility of the ICTV. The suggested convention of using lowercase for these names has been upheld.

Change Made: Described in lines 36-38 and 230-250

Comment 2: For grammatical accuracy, please revise lines 25–26 to either: "This study presents a comprehensive comparative genomic analysis and global distribution map using…." or "This study presents comprehensive comparative genomic analyses and a global distribution map using …".

Response 2: This suggestion is well appreciated and updated accordingly.

Change Made: Described in lines 24-25

Comment 3: Please revise lines 42-44: Furthermore, we hypothesized specific vector transmission: Opunvirus, Welwivirus, and Topilevirus are transmitted via treehopper species, whereas Citlodavirus and Eragrovirus are transmitted via leafhopper species.

Response 3: This suggestion is well appreciated and updated accordingly.

Change Made: Described in lines 42-44

Comment 4: Line 45: Consider changing "with" to "and" in the phrase "plant viruses with their transmission vectors..."

Response 4: This suggestion is well appreciated and updated accordingly.

Change Made: Described in line 45

Comment 5: Line 54: "tomatoes production" should be corrected to "tomato production".

Response 5: This suggestion is well appreciated and updated accordingly.

Change Made: Described in line 57

Comment 6: For lines 67-69, This emergence of new virus species and genera with modified virulence overcomes the host resistance gene and surveillance through the increased adaptation rate of the virus to environmental factors and susceptible host plants [15-17]. Suggested revision: The emergence of new virus species and genera with modified virulence can overcome host resistance genes and challenge surveillance due to the virus's increased adaptation to environmental factors and susceptible host plants [15-17]"

Response 6: This suggestion is well appreciated and updated accordingly.

Change Made: Described in lines 70-72

Comment 7: Lines 74-75: "Their respective studies were mainly on begomoviruses and a few other genera." Can be changed to "These studies mainly focused on begomoviruses and a few other genera."

Response 7: This suggestion is well appreciated and updated accordingly.
Change Made: Described in lines 77-78

Comment 8: It should be noted that the phrase on lines 83–84 remains incomplete. The verb "is essential" was missed during the previous revision. The corrected sentence "Understanding the diversity and evolution of both new and previously reported species is essential to support surveillance efforts against emerging diseases threatening food security."

Response 8: This suggestion is well appreciated and updated accordingly.

Change Made: Described in lines 86-88

Comment 9: Lines 96-97: "The diversity of these genera varies from one to another" Can be rephrase as 'The genetic diversity among these genera varies."

Response 9: This suggestion is well appreciated and updated accordingly.

Change Made: Described in lines 99-100

Comment 10: Lines 103-106: Suggested revision for more fluent wording" Sequences for Mastrevirus and Begomovirus, which had a large number of available sequences, were grouped into batches for parallel computation, and sampling was performed using ≥80% pairwise similarity. Similarly, Bandoo et al. [28] adopted 80% pairwise identity when selecting members of Begomovirus.

Response 10: This suggestion is well appreciated and updated accordingly.

Change Made: Described in lines 106-109

Comment 11: Lines 125-126: "This is to avoid graphic clutter, which will subsequently simplify the data visualization." Change to "This approach minimizes graphic clutter and facilitates data visualization."

Response 11: This suggestion is well appreciated and updated accordingly.

Change Made: Described in lines 128-129

Comment 12: Lines 139-141: "The integrated methods in RDP5 that were used to perform the preliminary scan for possible recombination in the aligned virus sequences are Chimaera [35], Bootscan [36–37], Geneconv [21], SisterScan [38], Maximum Chi Square [35, 39], and 3Seq [40]." can be rephrase as "The following integrated methods in RDP5 were used to perform a preliminary scan for possible recombination in the aligned virus sequences: Chimaera [35], Bootscan [36–37], Geneconv [21], SisterScan [38], Maximum Chi Square [35, 39], and 3Seq [40]."

Response 12: This suggestion is well appreciated and updated accordingly.

Change Made: Described in lines 141-143

Comment 13: Lines 147-151: "The analysis of the pairwise identity for the sampled genomes revealed the inter-genera pairwise identity across the monopartite of the entire genera of the

Geminiviridae and the Begomovirus DNA-A to be ≥40%….." can be rephrase as "Analysis of pairwise identity for the sampled genomes revealed that inter-genera identity among monopartite geminiviruses and Begomovirus DNA-A is ≥40% (Fig. 1), corresponding to a maximum genetic variation of 60%. Similarly, interspecies pairwise identity across the complete genomes of Begomovirus DNA-B is ≥41% (Fig. 2), corresponding to a maximum genetic variation of 59%".
Response 13: This suggestion is well appreciated and updated accordingly.

Change Made: Described in lines 149-152

Comment 14: 185-186: "Alternatively, few species of Begomovirus, including unclassified Begomovirus and Mastrevirus, clusters outside their respective genus members." can be rephrase as "However, a few Begomovirus species, including unclassified Begomovirus and Mastrevirus, cluster outside their respective genera."
Response 14**:** This suggestion is well appreciated and updated accordingly.

Change Made: Described in lines 184-185

Comment 15: Lines 208-209: "Interestingly, the species of Capulavirus was not among the entire viruses in the BLAST results." can be rephrase as "Interestingly, no Capulavirus species were detected in the BLAST results."

Response 15: This suggestion is well appreciated and updated accordingly.

Change Made: Described in lines 208-209

Comment 16: Lines 223-224: In the sentence "The virus nucleotide sequences and amino acid sequences of the coat protein have the highest percentage pairwise identity of 91.3% and 99.2%, respectively, to Corchorus yellow vein mosaic virus (CoYV) accessions KX513862 and AGG18212, respectively," the second 'respectively' is redundant. It can be removed: "…..have the highest percentage pairwise identity of 91.3% and 99.2% to Corchorus yellow vein mosaic virus (CoYV) accessions KX513862 and AGG18212, respectively".
Response 16**:** This suggestion is well appreciated and updated accordingly.

Change Made: Described in lines 223-225

Comment 17: Line 228: Since the abbreviation "CoYV" has already been introduced, it is not necessary to write the full name "Corchorus yellow vein mosaic virus" again. Please correct to "CoYV".

Response 17: This suggestion is well appreciated and updated accordingly.

Change Made: Described in line 228

Comment 18: Line 242: Importantly, CP emerged as the key genomic region distinguishing Begomovirus species from both Maldovirus members and Begomovirus spathoglottis 1 and 2 (Fig 11)." Consider revising to "The CP was identified as the primary genomic region distinguishing Begomovirus species from both Maldovirus members and begomovirus spathoglottis 1 and 2 (Fig 11)."

Response 18: This suggestion is well appreciated and updated accordingly.

Change Made: Described in lines 242-243

Comment 19: Lines 254-255: "Interspecies recombination was detected in higher numbers in Begomovirus; many species of this genus had recombinant sequences of other species in their genomes." Can be revised to "Interspecies recombination was more frequent in Begomovirus, with many species containing recombinant sequences derived from other species."
Response 19: This suggestion is well appreciated and updated accordingly.

Change Made: Described in lines 255-256

Comment 20: Line 329: The phrase "Contrarily, the CP of a few species…" should be revised. I recommend replacing "Contrarily" with "In contrast," which is more appropriate.

Response 20: This suggestion is well appreciated and updated accordingly.

Change Made: Described in line 330
